# Treatment of Night Sweats in Consumption

**Published:** 1869-02

**Authors:** 


### SELECTED ITEMS.

TREATMENT OF NIGHT-SWEATS IN CONSUMPTION.—Powdered borax drachms vss, washed sulphur 1 oz., sub-nitrate of bismuth, drachms iss.; divide into forty powders, one to be given every two hours (twelve a day.) Four or five days of treatment will suspend or much diminish this troublesome and exhausting symptom, and give much relief to the patient.—*Rodolphe.*

